# *Corynebacterium pseudogenitalium* Urinary Tract Infection

**DOI:** 10.3201/eid1202.050950

**Published:** 2006-02

**Authors:** Gérard Vedel, Gaëlle Toussaint, Philippe Riegel, Jean-Luc Fouilladieu, Annick Billöet, Claire Poyart

**Affiliations:** * Groupe Hospitalier Cochin Saint-Vincent-de-Paul La Roche-Guyon, Paris, France;; † Université Paris René Descartes, Paris, France;; ‡ Université Louis-Pasteur, Strasbourg, France

**Keywords:** Corynebacteria, urolithiasis, 16S rRNA, letter

**To the Editor:** A 64-year-old man was admitted to the urology department of Cochin Hospital in Paris, France, for acute urinary retention. He had a history of recurrent urolithiasis and undocumented urinary tract symptoms. At admission, a urethral catheter was inserted, and a plain radiograph showed 2 bladder stones and milk of calcium calcifications. Three days later, he underwent extracorporeal shock wave lithotripsy treatment, and empiric antimicrobial drug therapy with intravenous ceftriaxone, 1 g/day for 3 days, was administered. Three consecutive urinalyses showed a pH of 9, crystals of struvite, hematuria, and 10^5^ leukocytes/mL. Gram-positive rods with rounded ends and nonparallel sides, arranged in palisades or in V shapes, were observed, which suggested the presence of corynebacteria. Urine cultures were positive and yielded a pure culture of 10^5^ CFU/mL of *Corynebacterium* spp.

The isolated strain showed slight growth after 24 or 48 h of incubation on horse blood agar medium but abundant growth on the same medium containing 1% Tween 80 under aerobic conditions (5% CO_2_). Colonies were white, opaque, smooth, convex, and nonhemolytic. This lipid-requiring strain was catalase positive and strongly urease positive. Testing with the API-Coryne strip (bioMérieux, Marcy l'Etoile, France) showed that the strain was nitrate-reduction positive and produced acid from glucose, ribose, sucrose, and maltose. However, this strain, which was designated CCH052683, did not hydrolyze gelatin or esculin. It was identified as *Corynebacterium* group F1 (the corresponding numeric profile of the gallery API-Coryne was 3001325).

The strain was correctly identified to the species level as *Corynebacterium pseudogenitalium* by using polymerase chain reaction and sequencing 16 rRNA as previously described (*1**,**2*). Comparison of 785 nucleotides (546–1,331) gave a 16S rDNA similarity value of 99.9% between the sequences of the isolated strain and *C. pseudogenitalium* ATCC 33039/NCTC11860 (European Molecular Biology Laboratory accession no. X81872).

The strain was sensitive to penicillin, ampicillin, gentamicin, rifampin, vancomycin, teicoplanin, tetracycline, sulfamethoxazole, trimethoprim, fusidic acid, ciprofloxacin, and norfloxacin and resistant to erythromycin, lincomycin, and nitrofurantoin. Ceftriaxone was replaced by norfloxacin (400 mg twice a day) for 1 month. The patient improved and remained healthy 6 months after therapy.

Nondiphtheric corynebacteria are of increasing importance. They have been observed in human specimens, and many new taxa of coryneform bacteria have been described (*3*). Interest in their taxonomy is increasing, and molecular, phenotypic, and biochemical analyses have resulted in the reclassification of this genus (*3*). *C. pseudogenitalium* was described in 1979 by Furness et al. (*4*) for lipophilic corynebacteria isolated from urinary tract and was not considered a pathogen, in contrast to *C. genitalium*. However, these 2 species were not included in the official list of recognized species.

*C. pseudogenitalium* was divided into 5 types based on biochemical patterns, and strains of the type C-5 were differentiated from other types on the basis of urease production. The biochemical and physiologic characteristics of this C-5 type were similar of those of the coryneform group F-1 described by the Centers for Disease Control and Prevention (CDC). In 1995, a comprehensive study on lipophilic corynebacteria demonstrated by DNA-DNA hybridization the similarity between a reference strain of *C. pseudogenitalium* type C-5 and reference strains of the CDC coryneform group F-1 (*1*). The CDC group F-1 make up 2 genomic groups at the species level. As shown by 16S rDNA gene comparisons, isolate CCH052683 belongs to the genomic group, including a reference strain of *C. pseudogenitalium* type C-5 ATCC 33039 (CCUG 27540, sequence X81872) and a reference strain of CDC group F-1 (CDC G4330, sequence X81905) (Figure). The other genomic group of CDC group F-1 is represented by strain CDC G5911 (sequence X81904). The molecular genetic investigations identified our isolate as *C. pseudogenitalium* and placed it in 1 of the 2 genomic groups of CDC group F-1, which cannot be differentiated by biochemical tests (*1*).

**Figure:**
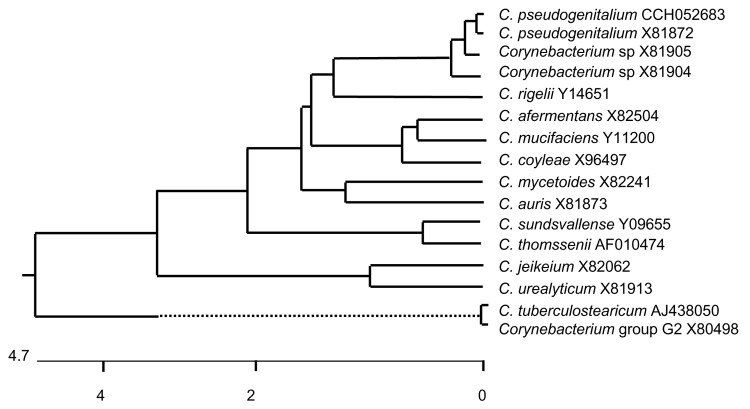
Unrooted tree showing phylogenetic relationships of *Corynebacterium pseudogenitalium* CCH052683 and other members of the genus *Corynebacterium*. The tree was constructed by using the DNAstar program (DNAstar Inc., Madison, WI, USA) (Clustal method) and based on a comparison of 785 (546–1,331) nucleotides. European Molecular Biology Laboratory sequence accession numbers are shown. The scale bar shows the percentage sequence divergence. Dotted line indicates a distant phylogenetic group for which the scale is not applicable.

The pathogenicity of this bacterium was associated with strong urease activity. This activity is similar to that of other urease-positive microorganisms, such as *C. urealyticum* and *Proteus* spp (*5**,**6*), which infect the urinary tract. Unfortunately, the bladder stones were not analyzed after extracorporeal shock wave lithotripsy treatment. The *C. pseudogenitalium* isolate was sensitive to most antimicrobial drugs, particularly β-lactams, aminoglycosides, and quinolones. Thus, urinary tract infections caused by this species of bacteria respond more readily to treatment than those caused by multidrug-resistant *C. urealyticum* (*3*).

In conclusion, we show that *C. pseudogenitalium* (CDC coryneform group F-1) can cause urinary tract infection (*7*) and produce urease, and like *C. urealyticum*, cause stone formation in humans. Thus, urease-positive microorganisms isolated by urinalysis that shows urinary alkalinization and struvite and pyuria crystallization should be considered pathogenic. Our results also confirm the difficulty in phenotypic identification of these strains and the need to use a molecular approach to identify coryneform bacteria with clinical relevance.
